# Early Changes in B and Plasma Cell Subsets and Traditional Serological Markers as Predictors of SRI-4 Response to Therapy in Systemic Lupus Erythematosus

**DOI:** 10.3389/fmed.2022.852162

**Published:** 2022-04-28

**Authors:** Ioannis Parodis, Alvaro Gomez, Julius Lindblom, Jun Weng Chow, Andrea Doria, Mariele Gatto

**Affiliations:** ^1^Division of Rheumatology, Department of Medicine Solna, Karolinska University Hospital, Karolinska Institutet, Stockholm, Sweden; ^2^Department of Rheumatology, Faculty of Medicine and Health, Örebro University, Örebro, Sweden; ^3^Unit of Rheumatology, Department of Medicine, University of Padua, Padua, Italy

**Keywords:** systemic lupus erythematosus, biomarkers, prediction, B cells, plasma cells, B lymphocyte, belimumab, biologics

## Abstract

**Objective:**

With the premise of the hypothesis that early biological responses to therapy for active systemic lupus erythematosus (SLE) portend later clinical improvements, we studied changes in B cell subsets and traditional serological markers in relation to clinical response to standard therapy (ST) with or without the addition of belimumab.

**Patients and Methods:**

We analyzed data from the BLISS-76, BLISS-SC, and BLISS Northeast Asia trials (*N* = 1712). Circulating CD19^+^ B cell subsets were determined by flow-cytometry. We studied associations of relative to baseline percentage changes in circulating B and plasma cell subsets, anti-dsDNA antibody levels and complement levels with SLE Responder Index (SRI)-4 response after 52 weeks of treatment. Changes occurring through week 8 were deemed “rapid,” through week 24 “early,” and thereafter “delayed”.

**Results:**

In the analysis of the entire cohort, SRI-4 responders showed more prominent decreases from baseline through week 52 in CD19^+^CD20^+^CD27^–^ naïve B cells (median change: −61.2% versus −50.0%; *P* = 0.004), CD19^+^CD20^–^CD27*^bright^* plasmablasts (−44.9% versus −33.3%; *P* = 0.011), and CD19^+^CD20^–^CD138^+^ long-lived plasma cells (−48.2% versus −37.1%; *P* = 0.024), and a more prominent rapid (+92.0% versus +66.7%; *P* = 0.002) and early (+60.0% versus +49.5%; *P* = 0.033) expansion of CD19^+^CD20^+^CD27^+^ memory B cells than non-responders. More prominent rapid reductions in anti-dsDNA (−14.8% versus −8.7%; *P* = 0.043) and increases in C3 (+4.9% versus +2.1%; *P* = 0.014) and C4 levels (+11.5% versus +8.3%; *P* = 0.017) were documented in SRI-4 responders compared with non-responders among patients who received add-on belimumab, but not among patients who received non-biological ST alone.

**Conclusion:**

SRI-4 responders showed a more prominent rapid expansion of memory B cells and more prominent delayed reductions in naïve B cells, plasmablasts and long-lived plasma cells. Moreover, clinical response to belimumab was associated with preceding more prominent reductions of anti-dsDNA and increases in C3 and C4 levels. Monitoring biological changes may prove useful in SLE patient surveillance and early treatment evaluation.

## Introduction

Systemic lupus erythematosus (SLE) is a chronic, inflammatory, autoimmune disease that predominantly affects women during their fertile age and is characterized by immense heterogeneity in clinical presentation (1). The treatment of SLE mainly consists of antimalarial agents, glucocorticoids, non-biological disease modifying anti-rheumatic drugs and since recently biological agents (2). The monoclonal antibody belimumab that selectively binds to the soluble counterpart of B cell activating factor (BAFF; also known as B lymphocyte stimulator, BLyS) is licensed for SLE treatment since 2011 (3), and for active lupus nephritis since 2021 (4). The efficacy of belimumab in inducing disease control and reducing the risk of disease flares has been documented in multiple clinical trials and real-life observational studies (5–18), including documentation of its long-term use (19), lending indirect corroboration to the important role of B cells in SLE pathogenesis (1).

Given its mode of action, belimumab is expected to impede the survival and differentiation of B cells, especially in their early stages, as shown in previous research (20–23). Declining counts of B cells could therefore be expected to portend good responses to belimumab therapy, in a similar fashion to successful B cell depletion heralding good clinical responses to rituximab (24, 25). In a real-life observational study of 23 patients with SLE, immunological responses upon commencement of belimumab therapy preceded overt clinical improvements, and low B cell counts were associated with favorable treatment outcome (21). Taken together, we hypothesized that early biological changes upon commencement of belimumab therapy that are consistent with abatement of B cell activity might portend clinical improvements at later timepoints.

Hence, the aim of the present study was to investigate alterations in B and plasma cell subsets as well as selected traditional serological markers in relation to clinical response to therapy for active SLE. More specifically, we investigated B and plasma cell alterations in relation to response to non-biological standard therapy (ST) with or without addition of belimumab, utilizing data from three phase III clinical trials of belimumab in SLE (6–8). Identification of reproducible biological changes that occur soon after treatment commencement and precede clinical response could introduce a novel concept in surveillance of SLE patients, lending promise in early treatment evaluation and thus contributing to a more person-centered therapeutic decision-making and a better use of economic resources.

## Materials and Methods

### Study Population

We designed a *post-hoc* analysis of data from three multicentre, randomized, double-blind, placebo-controlled phase III clinical trials of belimumab i.e., BLISS-76 (NCT00410384) (6), BLISS-SC (NCT01484496) (7), and BLISS Northeast Asia (NEA; NCT01345253) (8). A total of 1712 patients (819, 833, and 60, respectively) were deemed eligible for analysis, based on availability of flow cytometry data for B and plasma cell subsets, along with data on selected serological markers. In these trials, belimumab or placebo was administered intravenously (BLISS-76 and BLISS-NEA; at day 0, 14, and 28 from baseline, and thereafter every 4th week through week 48 in BLISS-NEA and through week 72 in BLISS-76) or subcutaneously (BLISS-SC; belimumab 200 mg or placebo weekly through week 52) on top of non-biological ST, the latter including antimalarial agents, glucocorticoids, immunosuppressants (mainly mycophenolate mofetil, methotrexate, and azathioprine), or combinations thereof.

Briefly, patients were required to have a Safety of Estrogens in Lupus Erythematosus National Assessment - Systemic Lupus Erythematosus Disease Activity Index (SELENA-SLEDAI) (26) score ≥6 (BLISS-76) or ≥8 (BLISS-SC and BLISS-NEA) and had to be autoantibody positive, defined as antinuclear antibody titers ≥1:80 and/or anti-double stranded (ds)DNA levels ≥30 IU/mL. The main exclusion criteria were similar across the three trials and encompassed severe active lupus nephritis or neuropsychiatric SLE, pregnancy, previous treatment with B cell targeting therapy, intravenous cyclophosphamide within 6 months prior to enrollment, and intravenous immunoglobulin, other biologics, prednisone (>100 mg/day) or plasmapheresis within 3 months prior to enrollment. All patients had been on stable doses of non-biological ST for at least 30 days prior to belimumab or placebo commencement (baseline). Gradual restrictions regarding allowance in changes in the background immunosuppressive and antimalarial therapy were imposed during the study periods, as well as restrictions regarding glucocorticoid intake. The similar design across the three trials facilitated pooling of data prior to analysis.

### Definition of Clinical Response

The primary efficacy endpoint was common across the three trials i.e., the proportion of clinical responders at week 52, with clinical response being defined as attainment of the SLE Responder Index (SRI)-4 criteria (27). SRI-4 response required (i) *a* ≥4 point reduction in the SELENA-SLEDAI score compared with baseline i.e., resolution of at least one SLE disease manifestation, (ii) no new British Isles Lupus Assessment Group (BILAG) (28). A domain score or no more than one new BILAG B score i.e., no significant flares or worsening of the condition, and (iii) no more than a 30% increase in the Physician’s Global Assessment (PGA) score (measured on a 0–3 scale) (26), and served as the definition of clinical response in the present analysis.

### B Cell Subsets and Serological Markers

Peripheral B and plasma cell subsets were determined with flow cytometry performed within the frame of the BLISS trials (6–8) and subcategorised into total peripheral CD19^+^CD20^+^ B cells, CD19^+^CD20^+^CD69^+^ activated B cells, CD19^+^CD20^+^CD27^–^ naïve B cells, CD19^+^CD20^+^CD27^+^ memory B cells, CD19^+^CD20^–^CD27*^bright^* plasmablasts, CD19^+^CD20^+^CD138^+^ short-lived plasma cells, CD19^+^CD20^–^CD138^+^ long-lived plasma cells and CD19^+^CD27*^bright^*CD38*^bright^* SLE-associated plasma cells, as previously described (20, 29, 30). Serum levels of anti-dsDNA, C3 and C4 were determined within the frame of the BLISS trials (6–8) and were made available through the Clinical Study Data Request (CSDR) consortium.

We analyzed percentages of relative to baseline (i.e., treatment commencement) changes in B and plasma cell subsets as well as in serum levels of anti-dsDNA, C3, and C4 that occurred through week 8, 24, and 52. Changes occurring through week 8 were deemed rapid, changes occurring through week 24 were deemed early, and changes occurring thereafter were referred to as delayed. We next investigated associations between changes in B cell or plasma cell subsets or changes in serological markers and SRI-4 response at week 52 in the entire patient population, in patients who received add-on belimumab, and in patients who received non-biological ST alone.

### Ethics

Data from the BLISS-76, BLISS-SC and BLISS-NEA trials were made available by GlaxoSmithKline (Uxbridge, United Kingdom) through the CSDR consortium. The trial protocols were approved by regional ethics review boards at all participating centers and complied with the ethical principles of the Declaration of Helsinki. Written informed consent was obtained from all study participants prior to enrollment. The present study was approved by the Swedish Ethical Review Authority (2019-05498).

### Statistics

Descriptive statistics are reported as means and standard deviations or medians and interquartile ranges for continuous variables. Frequencies are reported for categorical variables. Values (relative to baseline percentage change) above the 97.5th percentile were treated as extreme values and set to a same max value (equal to the 97.5th percentile) for each cell variable. Comparisons of distributions of relative to baseline changes between groups (SRI-4 responders versus non-responders, and patients who received belimumab versus placebo) were conducted using the non-parametric Mann–Whitney *U* test. *P*-values below 0.05 were deemed statistically significant. All analyses were performed using the R version 4.01 software (R Foundation for Statistical Computing, Vienna, Austria). The GraphPad Prism software version 9 (La Jolla, CA, United States) was used for the preparation of graphs.

## Results

### Patient Characteristics

Demographics, clinical and serological data of the patients including comparisons between SRI-4 responders and non-responders are reported in Table 1. In the pooled dataset, 818/1712 patients (47.8%) attained SRI-4 at week 52. Baseline B and plasma cell data including comparisons between patients who attained and patients who did not attain clinical response at week 52 are reported in Table 2, stratified by trial to account for batch variations in flow cytometry readouts across the BLISS trials.

**TABLE 1:** Characteristics of SRI-4 responders versus non-responders at week 52 in the pooled BLISS study population.

	All patients	SRI-4	No SRI-4	*P*-value
		
	*N* = 1712	*N* = 818	*N* = 894	
**Patient characteristics**				
Age at baseline (years)	39.3 ± 11.9	38.9 ± 11.8	39.6 ± 12.0	0.223
Female sex	1605 (93.8%)	770 (94.1%)	835 (93.4%)	0.532
**Ancestry**				
Asian	269 (15.7%)	129 (15.8%)	140 (15.7%)	0.950
Black/African American	203 (11.9%)	76 (9.3%)	127 (14.2%)	**0.002**
Indigenous American*	170 (9.9%)	103 (12.6%)	67 (7.5%)	**<0.001**
White/Caucasian	1070 (62.5%)	510 (62.3%)	560 (62.6%)	0.901
**Clinical data**				
SLE duration at baseline (years)	5.1 (1.6-10.6)	4.6 (1.4-9.6)	5.6 (1.9-11.4)	**0.001**
**Treatment at baseline**				
Glucocorticoids	1403 (82.0%)	690 (84.4%)	713 (79.8%)	**0.013**
AMA^†^	1097 (64.1%)	537 (65.6%)	560 (62.6%)	0.195
Immunosuppressants^‡^	881 (51.5%)	387 (47.3%)	494 (55.3%)	**0.001**
Azathioprine	335 (19.6%)	159 (19.4%)	176 (19.7%)	0.897
Methotrexate	248 (14.5%)	102 (12.5%)	146 (16.3%)	**0.023**
Mycophenolate mofetil or sodium	243 (14.2%)	100 (12.2%)	143 (16.0%)	**0.026**
Trial intervention				
Placebo	575 (33.6%)	232 (28.4%)	343 (38.4%)	<**0.001**
Belimumab	1137 (66.4%)	586 (71.6%)	551 (61.6%)	<**0.001**
i.v. 1 mg/kg	271 (15.8%)	110 (13.4%)	161 (18.0%)	**0.010**
i.v. 10 mg/kg	312 (18.2%)	136 (16.6%)	176 (19.7%)	0.101
s.c. 200 mg	554 (32.4%)	340 (41.6%)	214 (23.9%)	<**0.001**
**Serological markers at baseline**				
C3; mg/dL	95.5 (74.0-118.0)	96.0 (76.0–118.0)	95.0 (72.0–118.0)	0.298
C4; mg/dL	15.0 (9.0-22.0)	15.0 (9.0–22.0)	14.0 (8.0–22.0)	0.095
Anti-dsDNA; IU/mL (all patients)	95.0 (29.0–287.8)	91.5 (29.0–269.3)	97.5 (29.0–321.5)	0.366
Anti-dsDNA; IU/mL (patients positive at baseline)	167.0 (89.0–495.8); *N* = 1170	149.0 (82.8–438.5); *N* = 570	189.5 (97.0–527.5); *N* = 600	**0.023**

*Data are presented as number (percentage), mean ± standard deviation, or median (interquartile range), as appropriate. In case of missing values, the total number of patients with available data is indicated. Statistically significant P-values are in bold.*

*Alaska Native or American Indian from North, South, or Central America.

*^†^Hydroxychloroquine, chloroquine, mepacrine, mepacrine hydrochloride or quinine sulfate.*

*^‡^Azathioprine, cyclosporine, oral cyclophosphamide, leflunomide, methotrexate, mizoribine, mycophenolate mofetil, mycophenolate sodium, or thalidomide. AMA, antimalarial agents; C3, complement component 3; C4, complement component 4; i.v., intravenous; s.c., subcutaneous; SLE, systemic lupus erythematosus; SRI-4, SLE Responder Index 4.*

**TABLE 2:** B cell subset counts at baseline in SRI-4 responders versus non-responders at week 52 in the BLISS-76, BLISS-SC, and BLISS Northeast Asia study population.

B cell subsets	All patients	SRI-4	No SRI-4	*P*-value

**BLISS-76**				

	***N* = 819**	***N* = 320**	***N* = 499**	
CD19^+^CD20^+^ (x10^3^/mL)	91.5 (43.0–176.0); *N* = 756	97.0 (42.3–187.0); *N* = 292	88.0 (43.3–166.8); *N* = 464	0.306
CD19^+^CD20^+^CD27^+^ (x10^3^/mL)	14.0 (6.0–27.0); *N* = 756	15.0 (7.0–27.0); *N* = 292	14.0 (6.0–26.0); *N* = 464	0.191
CD19^+^CD20^+^CD69^+^ (/mL)	2096.5 (938.3–4350.8); *N* = 744	2230.0 (721.0–4408.0); *N* = 287	2071.0 (1017.0–4322.0); *N* = 457	0.631
CD19^+^CD20^+^CD27^–^ (x10^3^/mL)	75.0 (33.0–143.0); *N* = 756	81.0 (32.0–151.8); *N* = 292	72.0 (34.0–134.3); *N* = 464	0.377
CD19^+^CD20^+^CD138^+^ (/mL)	819.0 (334.0–1811.5); *N* = 749	832.0 (315.0–1772.0); *N* = 289	802.5 (345.3–1820.0); *N* = 460	0.654
CD19^+^CD20^–^CD138^+^ (/mL)	482.5 (211.0–1067.3); *N* = 748	483.5 (199.0–1028.0); *N* = 288	481.0 (220.0–1098.0); *N* = 460	0.499
CD19^+^CD20^–^CD27*^brt^* (/mL)	299.0 (115.0–705.0); *N* = 747	350.0 (115.0–713.0); *N* = 287	282.0 (115.5–685.0); *N* = 460	0.224
CD19^+^CD27*^brt^*CD38*^brt^* (/mL)	306.0 (116.0–701.8); *N* = 754	315.0 (121.0–760.0); *N* = 291	301.0 (113.0–677.0); *N* = 463	0.296

**BLISS–SC**				

	***N* = 833**	***N* = 475**	***N* = 358**	

CD19^+^CD20^+^ (x10^3^/mL)	106.5 (56.0–196.0); *N* = 808	106.5 (59.0–198.5); *N* = 462	106.5 (53.0–193.3); *N* = 346	0.589
CD19^+^CD20^+^CD27^+^ (x10^3^/mL)	14.0 (7.0–29.0); *N* = 808	14.0 (7.0–30.0); *N* = 462	14.0 (7.0–25.0); *N* = 346	0.320
CD19^+^CD20^+^CD69^+^ (/mL)	80.0 (33.0–200.5); *N* = 808	87.5 (34.0–216.0); *N* = 462	74.5 (31.0–176.3); *N* = 346	0.131
CD19^+^CD20^+^CD27^–^ (x10^3^/mL)	89.0 (43.0–167.0); *N* = 808	88.0 (45.8–168.5); *N* = 462	91.0 (41.8–166.0); *N* = 346	0.756
CD19^+^CD20^+^CD138^+^ (/mL)	53.0 (20.0–126.8); *N* = 808	52.5 (20.0–131.3); *N* = 462	55.0 (19.0–126.3); *N* = 346	0.925
CD19^+^CD20^–^CD138^+^ (/mL)	202.0 (67.3–504.8); *N* = 808	194.5 (67.0–504.3); *N* = 462	212.0 (70.5–508.3); *N* = 346	0.498
CD19^+^CD20^–^CD27*^brt^* (/mL)	2000.0 (1000.0–4000.0); *N* = 808	2000.0 (1000.0–4000.0); *N* = 462	2000.0 (1000.0–4000.0); *N* = 346	0.158
CD19^+^CD27*^brt^*CD38*^brt^* (/mL)	1732.5 (738.0–3933.5); *N* = 808	1802.5 (753.8–3979.3); *N* = 462	1620.5 (705.8–3905.3); *N* = 346	0.252

**BLISS Northeast Asia**				

	***N* = 60**	***N* = 23**	***N* = 37**	

CD19^+^CD20^+^ (x10^3^/mL)	52.5 (22.8–96.8); *N* = 54	49.0 (28.0–89.0); *N* = 21	54.0 (21.5–108.0); *N* = 33	0.852
CD19^+^CD20^+^CD27^+^ (x10^3^/mL)	7.3 (3.7–10.6); *N* = 55	7.5 (4.2–13.2); *N* = 21	7.0 (3.6–10.6); *N* = 34	0.716
CD19^+^CD20^+^CD69^+^ (/mL)	101.3 (45.9–183.0); *N* = 55	115.8 (51.9–228.8); *N* = 21	91.3 (45.2–180.0); *N* = 34	0.533
CD19^+^CD20^+^CD27^–^ (x10^3^/mL)	39.7 (18.6–87.5); *N* = 55	39.4 (25.3–82.4); *N* = 21	43.1 (17.3–90.6); *N* = 34	0.986
CD19^+^CD20^+^CD138^+^ (/mL)	108.2 (58.1–258.1); *N* = 55	82.4 (57.5–169.6); *N* = 21	114.1 (54.9–377.6); *N* = 34	0.446
CD19^+^CD20^–^CD138^+^ (/mL)	303.1 (174.5 –668.8); *N* = 55	233.8 (137.3–604.2); *N* = 21	340.2 (196.2–721.0); *N* = 34	0.188
CD19^+^CD20^–^CD27*^brt^* (/mL)	916.5 (262.8–2008.4); *N* = 55	696.7 (195.8–1319.2); *N* = 21	1037.0 (345.5–2951.4); *N* = 34	0.188
CD19^+^CD27*^brt^*CD38*^brt^* (/mL)	934.9 (264.7–2095.6); *N* = 55	741.8 (210.8–1451.0); *N* = 21	985.6 (405.0–2522.4); *N* = 34	0.260

*Data are presented as medians (interquartile range) of absolute counts. In case of missing values, the total number of patients with available data is indicated. P-values are derived from non-parametrical Mann–Whitney U tests. Statistically significant P-values are in bold. SC, subcutaneous; SRI-4, Systemic Lupus Erythematosus Responder Index 4.*

### B and Plasma Cell Kinetics in Relation to SRI-4 Response

**FIGURE 1:**
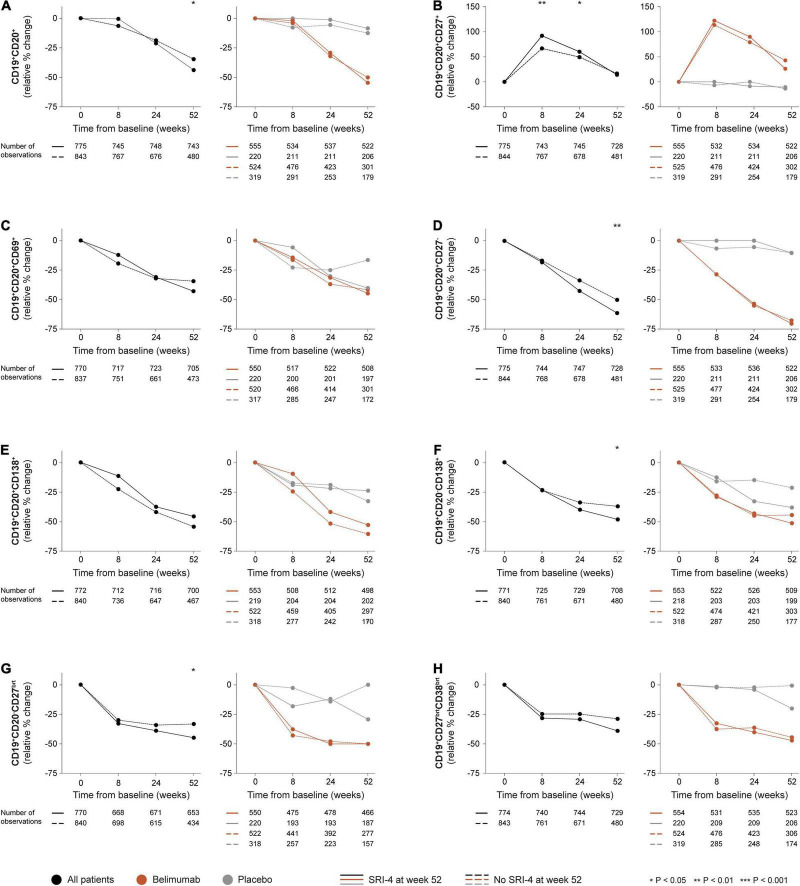
B and plasma cell subset alterations in relation to clinical response. The graphs delineate relative to baseline percentage changes in selected B cell and plasma cell subsets in patients who attained SRI-4 response at week 52 from baseline (continuous lines) and patients who did not (dashed lines). Comparisons between SRI-4 responders and non-responders were conducted for the entire population with available data (black lines), and after stratification into patients who received standard therapy plus belimumab (terracotta lines) and patients who received standard therapy alone (gray lines). *P*-values derived from non-parametric Mann–Whitney *U* tests. The number of patients with available data at each timepoint is indicated for each patient subgroup. SRI, Systemic lupus erythematosus Responder Index.

In the entire patient population i.e., all treatment arms, a more prominent relative to baseline decrease in CD19^+^CD20^+^ B cells was documented among SRI-4 responders compared with non-responders at week 52 (median change: −43.8% versus −34.7%; *P* = 0.023), but not at earlier timepoints (Figure 1A). A similar pattern was seen for CD19^+^CD20^+^CD27^–^ naïve B cells (−61.2% versus −50.0%; *P* = 0.004; Figure 1D), CD19^+^CD20^–^CD27*^bright^* plasmablasts (−44.9% versus −33.3%; *P* = 0.011; Figure 1G), and CD19^+^CD20^–^CD138^+^ long-lived plasma cells (−48.2% versus −37.1%; *P* = 0.024; Figure 1F), as well as in numerical but not statistically significant terms for CD19^+^CD20^+^CD69^+^ activated B cells (−43.0% versus −34.4%; *P* = 0.300; Figure 1C) and CD19^+^CD27*^bright^*CD38*^bright^* SLE-associated plasma cells (−38.9% versus −28.9%; *P* = 0.148; Figure 1H). By contrast, SRI-4 responders were characterized by a more prominent rapid (+92.0% versus +66.7%; *P* = 0.002) and early (+60.0% versus +49.5%; *P* = 0.033) expansion of CD19^+^CD20^+^CD27^+^ memory B cells compared with non-responders (Figure 1B), with a subsequent return toward baseline values through week 52, resulting in no discrepant change between SRI-4 responders and non-responders (+14.3% versus +16.7%; *P* = 0.988). Results are detailed in the online Supplementary Material.

In stratified analysis, differences in relative to baseline B and plasma cell changes between SRI-4 responders and non-responders did not reach statistical significance among patients who received add-on belimumab (any dose or administration route) or among patients who received ST alone (Figure 1).

CD19^+^CD20^+^ B cells showed more prominent reductions in patients who received belimumab compared with patients who received ST alone from week 24 onward, with similar patterns observed for CD19^+^CD20^+^CD27^–^ naïve B cells, CD19^+^CD20^–^CD27*^bright^* plasmablasts, and CD19^+^CD27*^bright^*CD38*^bright^* SLE-associated plasma cells from week 8 onward. The expanding-returning pattern for CD19^+^CD20^+^CD27^+^ memory B cells was only seen in patients who received belimumab, yielding significant differences compared with patients who received ST alone, yet irrespective of SRI-4 response (Figure 1 and Supplementary Material).

### Changes in Serological Markers in Relation to SRI-4 Response

**FIGURE 2:**
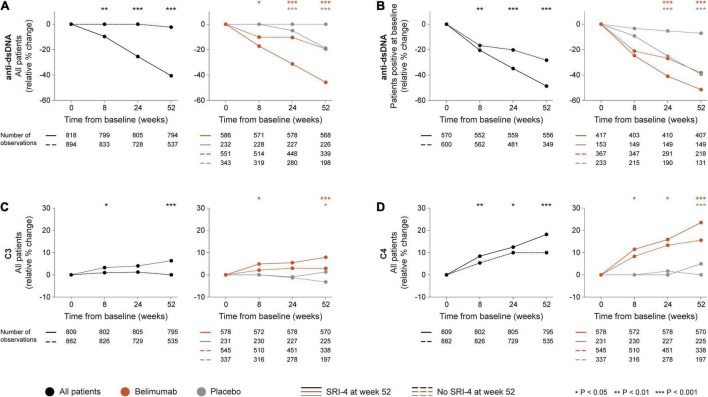
Changes in selected serological markers in relation to clinical response. The graphs delineate relative to baseline percentage changes in anti-dsDNA, C3, and C4 levels in patients who attained SRI-4 response at week 52 from baseline (continuous lines) and patients who did not (dashed lines). Comparisons between SRI-4 responders and non-responders were conducted for the entire population with available data (black lines), and after stratification into patients who received standard therapy plus belimumab (terracotta lines) and patients who received standard therapy alone (gray lines). For anti-dsDNA levels, a separate analysis for patients with positive anti-dsDNA levels (≥30 IU/mL) at baseline is also demonstrated. *P*-values derived from non-parametric Mann–Whitney *U* tests. The number of patients with available data at each time point is indicated for each patient subgroup. Anti-dsDNA: anti-double stranded DNA antibodies; C3: complement component 3; C4: complement component 4; SRI: Systemic lupus erythematosus Responder Index.

In the entire patient population, a more prominent relative to baseline decline in anti-dsDNA levels was documented in SRI-4 responders compared with non-responders as early as 8 weeks after therapy commencement (−8.3% versus 0.0%; *P* = 0.006). This difference persisted for changes in anti-dsDNA levels from baseline through week 24 (−21.8% versus 0.0%; *P* < 0.001) and week 52 (−34.8% versus −2.0%; *P* < 0.001; Figure 2A), and was also seen in the subgroup of patients with baseline anti-dsDNA levels above the threshold for positivity (≥30 IU/mL) from baseline through week 8 (−20.6% versus −16.7%; *P* = 0.005), week 24 (−34.8% versus −20.3%; *P* < 0.001) and week 52 (−48.7% versus −28.3%; *P* < 0.001; Figure 2B). Similarly, a more prominent increase in C3 levels was seen in SRI-4 responders compared with non-responders from baseline through week 8 (+3.3% versus +1.0%; *P* = 0.012) and week 52 (+6.3% versus 0.0%; *P* < 0.001; Figure 2C), as well as in C4 levels from baseline through week 8 (+8.5% versus +5.4%; *P* = 0.003), week 24 (+12.5% versus +10.0%; *P* = 0.017) and week 52 (+18.2% versus +10.0%; *P* < 0.001; Figure 2D).

In stratified analysis, differences in relative to baseline reductions in anti-dsDNA levels and increases in C3 and C4 levels were overall more prominent in patients who received add-on belimumab than in patients who received ST alone (Supplementary Material). Notably, during the rapid phase i.e., from baseline through week 8, we observed more prominent reductions in anti-dsDNA levels in patients who attained SRI-4 response compared with non-responders within the belimumab-treated population (−14.8% versus −8.7%; *P* = 0.043; Figure 2A), but not within patients who received ST alone. In a similar fashion, the rapid increases in C3 and C4 levels were more prominent in SRI-4 responders compared with non-responders in belimumab-treated patients (+4.9% versus +2.1%; *P* = 0.014; Figure 2C and +11.5% versus +8.3%; *P* = 0.017; Figure 2D, respectively), but not in patients who received ST alone.

## Discussion

We investigated alterations across different circulating B and plasma cell subsets as well as selected traditional serological markers upon treatment for active SLE and their associations with clinical response documented 52 weeks after treatment commencement. We demonstrated that CD19^+^CD20^+^ B cells decreased more prominently in responders than in non-responders to therapy, particularly CD19^+^CD20^+^CD27^–^ naïve B cells. Moreover, CD19^+^CD20^–^CD27*^bright^* plasmablasts and CD19^+^CD20^–^CD138^+^ long-lived plasma cells also decreased more prominently in clinical responders than in non-responders. However, this separation for both B cell and plasma cell subsets became significant only for the delayed follow-up phase i.e., for relative to baseline changes through week 52. By contrast, clinical responders showed a more prominent rapid expansion of CD19^+^CD20^+^CD27^+^ memory B cells compared with non-responders. While memory B cells tended to return toward baseline values thereafter, this separation between responders and non-responders was also present in the early phase i.e., from baseline through week 24. After stratification into active arm and placebo, it became evident that this expanding-returning pattern for memory B cells was induced by belimumab, a phenomenon that has been highlighted in previous studies (20, 21, 23).

Furthermore, we demonstrated that reductions in anti-dsDNA and increases in C3 and C4 levels distinguished clinical responders from non-responders during follow-up, with significant separations documented as early as 8 weeks after treatment initiation. After stratification into active arm and placebo, this separation remained significant for both belimumab-treated patients and patients who received ST alone at the evaluation of the delayed phase, but was only present in belimumab-treated patients during the rapid phase, suggesting that belimumab induces rapid and sustained changes in these serological markers, which are more prominent in patients who will show clinical response to treatment and could thus serve as useful markers in early treatment evaluation.

Several of these findings warrant further discussion. In the first place, belimumab was shown to induce rapid and sustained decreases in plasma cell subsets, with a clear separation from the placebo group irrespective of response to treatment. This finding is of interest in light of previous literature that has shown rather delayed or no plasma cell affection by belimumab therapy (21–23). This discrepancy may at least partly be due to the large SLE population in the present study which amplified the power in statistical calculations, and to some extent due to the detailed characterization of plasma cells into different subsets.

Another point of interest was the expanding-returning pattern of memory B cells, which herein showed ability to separate between clinical responders and non-responders. This is in line with our previous findings that a rapid expansion of memory B cells is associated with a lower probability of severe flare and renal flare (BR31a). After stratification into the belimumab and placebo arms, this expanding-returning pattern of memory B cells was only seen in patients treated with add-on belimumab, illustrating a phenomenon induced by belimumab that has been documented in several previous studies (20, 21, 23). While preservation of memory B cells upon belimumab therapy may be hypothesized to be due to the fact that their survival is not dependent on BAFF (32), the explanation underlying the rapid expansion and subsequent return of memory B cells has not been thoroughly elucidated. In fact, serum levels of a proliferation inducing ligand (APRIL), the tumour necrosis factor (TNF) ligand superfamily member that is most homologous to BAFF (33, 34), have been shown to decrease during belimumab therapy (13), suggesting that APRIL is to a larger extent consumed on its receptors on the surface of B cells in the milieu of a dearth of biologically active BAFF. Based on the known effects of BAFF on B cells (35) as well as early proof-of-concept studies on animals (36) and a phase II trial of belimumab (37), Stohl et al. speculated that the expanding-returning pattern of memory B cells may be a result of release from disrupted germinal centers where memory B cells reside, or a result of inhibition of their return to these lymphoid tissues, or a consequence of enhanced B cell differentiation from naïve to memory B cells (20). Findings from a recent study by Arends et al. suggested that this phenomenon may be due to secondarily disrupted lymphocyte trafficking owing to downregulated expression of genes coding for migration markers such as L-selectin (also known as CD62L) and intercellular adhesion molecule 2 (ICAM2; also known as CD102), which might prevent homing of lymphocytes to inflamed tissues and culminate in an abundance of memory B cells in the bloodstream (38). Collectively, this pattern of memory B cells during the rapid and early phases of belimumab therapy may not only have interest in terms of underlying biology, but also in terms of usefulness in the early evaluation of belimumab therapy where a lack of this pattern may signify lower probability of clinical response.

Another interesting finding was the ability of anti-dsDNA and complement level kinetics to separate between responders and non-responders as early as 8 weeks from treatment commencement, with a continuous and even more prominent separation during later timepoints. Importantly, while a delayed separation was present irrespective of the therapeutic regimen, rapid reductions of anti-dsDNA levels and rapid increases of C3 and C4 levels were more prominent in responders than in non-responders among belimumab-treated patients. These findings are in line with previous reports of biological changes preceding the overt clinical improvement induced by belimumab (21), and while changes in these serological markers might theoretically be expected to follow the kinetics of B cells, the demonstration that these traditional serological markers were more sensitive to change than B cell subsets and preceded B cell reductions and clinical response illustrates that their interrelationship is not always consequential. In this regard, it should also be noted that the choice of SRI-4 for the determination of clinical response may have magnified the impact of anti-dsDNA and complement level kinetics over B cell alterations since dsDNA binding and complement consumption are integral items of the SELENA-SLEDAI, one of the components of SRI-4, which is not the case for B cells.

It is important to clarify that monitoring early biological changes to portend therapeutic outcome should not be considered contradicting to baseline predictors, but complemental toward optimized person-centered surveillance. In fact, while serological status at baseline has been shown to predict the outcome of belimumab therapy in some studies (39, 40), this has not been consistent throughout the literature (13, 15, 41, 42). In a recent study that investigated selected autoantibodies and cytokines as predictors of response to belimumab therapy, early decreases in serum levels of interleukin (IL)-6 showed merit (43). Along the same lines, our study introduces the concept of rapid and early kinetics of selected markers, herein anti-dsDNA and complement levels in particular, as a complemental surveillance tool that may prove useful in early treatment evaluation.

This study has some limitations. Firstly, it was a *post-hoc* analysis of trials which were not designed to address the research question of the present study, which may have hampered the power in stratified statistical analyses. Secondly, the study participants comprised a selected SLE population with primarily musculoskeletal and mucocutaneous activity at baseline and excluded patients with severe active lupus nephritis and severe active central nervous system disease, which limits the generalizability of the findings to real-life SLE populations. Lastly, the characterization of B cell subsets and measurement of serological markers within the frame of three different trials may have introduced confounding due to batch effects, which limited us from studying absolute changes and necessitated investigation of changes relative to baseline. Nevertheless, the study encompassed a large number of patients that commenced therapy for active autoantibody positive SLE and were followed up in a structured manner within the frame of controlled phase III clinical trial programmes, ensuring diligent data collection and scarce occurrence of data missingness.

In summary, we demonstrated that SRI-4 responders showed a more prominent rapid expansion of memory B cells and more prominent delayed reductions in naïve B cells, plasmablasts and long-lived plasma cells. Moreover, clinical response established 1 year after commencement of belimumab therapy was preceded by more prominent rapid reductions of anti-dsDNA and more prominent rapid increases in C3 and C4 levels than in patients who did not respond to therapy. Our findings lend support for the usefulness of B and plasma cell kinetics as a complement to clinical features and traditional serological markers in treatment evaluation, and suggest that surveillance of anti-dsDNA and complement level kinetics may prove helpful in early evaluation of belimumab therapy.

## Data Availability Statement

The original contributions presented in the study are included in the article/Supplementary Material, further inquiries can be directed to the corresponding author.

## Ethics Statement

The studies involving human participants were reviewed and approved by the Swedish Ethical Review Authority. The patients/participants provided their written informed consent to participate in the BLISS trials.

## Author Contributions

IP and MG: study conception and design. IP, AG, JL, and JC: acquisition of data. IP, AG, JL, AD, and MG: analysis and interpretation of data. All authors were involved in the drafting of the manuscript or revising it critically for important intellectual content and approved the final version to be submitted for publication.

## Conflict of Interest

IP has received research funding and/or honoraria from Amgen, AstraZeneca, Aurinia Pharmaceuticals, Elli Lilly and Company, Gilead Sciences, GlaxoSmithKline, Janssen Pharmaceuticals, Novartis, and F. Hoffmann-La Roche AG. AD has received research funding and/or honoraria from AstraZeneca, Bristol-Myers Squibb, Celgene, Elli Lilly and Company, GlaxoSmithKline, Pfizer, and F. Hoffmann-La Roche AG. The remaining authors declare that the research was conducted in the absence of any commercial or financial relationships that could be construed as a potential conflict of interest.

## Publisher’s Note

All claims expressed in this article are solely those of the authors and do not necessarily represent those of their affiliated organizations, or those of the publisher, the editors and the reviewers. Any product that may be evaluated in this article, or claim that may be made by its manufacturer, is not guaranteed or endorsed by the publisher.
