# Supplementary material for: Early Changes in B and Plasma Cell Subsets and Traditional Serological Markers as Predictors of SRI-4 Response to Therapy in Systemic Lupus Erythematosus
Source: Front Med (Lausanne). 2022 Apr 28;9:852162. doi: 10.3389/fmed.2022.852162 (PMC9096349; doi:10.3389/fmed.2022.852162)
Supplement: Supplementary file 1 [file Data_Sheet_1.docx]

Supplementary Material

**Supplementary Table S1.** Relative to baseline percentage changes through week 8 in B cell subset counts and serum levels of serological markers in SRI-4 responders versus non-responders at week 52 in the pooled BLISS study population.

|  | **SRI-4** | **No SRI-4** | | | **P value** |
| --- | --- | --- | --- | --- | --- |
| **Entire patient cohort** (all treatment arms) | | | | | |
| **B cell subsets** |  | |  |  | |
| **CD19^+^CD20^+^** | -0.6 (-29.6−44.1) | -6.5 (-31.0−35.4) | | | 0.089 |
| **CD19^+^CD20^+^CD27^+^** | 92.0 (11.4−200.0) | 66.7 (-3.0−175.0) | | | **0.002** |
| **CD19^+^CD20^+^CD69^+^** | -12.3 (-68.4−109.1) | -19.5 (-64.6−106.7) | | | 0.930 |
| **CD19^+^CD20^+^CD27^-^** | -18.2 (-45.6−21.0) | -16.8 (-44.4−15.2) | | | 0.811 |
| **CD19^+^CD20^+^CD138^+^** | -11.4 (-67.6−109.3) | -22.6 (-69.7−81.4) | | | 0.223 |
| **CD19^+^CD20^-^CD138^+^** | -23.4 (-67.6−60.8) | -23.6 (-65.4−66.4) | | | 0.512 |
| **CD19^+^CD20^-^CD27^brt^** | -33.0 (-66.7−18.1) | -30.0 (-66.7−33.9) | | | 0.584 |
| **CD19^+^CD27^brt^CD38^brt^** | -28.2 (-60.4−50.0) | -24.8 (-63.2−50.4) | | | 0.944 |
| **Serological markers** |  |  | | |  |
| **C3** | 3.3 (-7.1−15.5) | 1.0 (-9.6−13.3) | | | **0.012** |
| **C4** | 8.5 (-4.8−28.6) | 5.4 (-9.1−22.3) | | | **0.003** |
| **anti-dsDNA** (all patients) | -8.3 (-34.4−0.0) | 0.0 (-28.0−0.4) | | | **0.006** |
| **anti-dsDNA** (patients positive at baseline) | -20.6 (-41.5−2.8) | -16.7 (-35.4−9.6) | | | **0.005** |
| **Belimumab** | | | | | |
| **B cell subsets** |  |  | | |  |
| **CD19^+^CD20^+^** | -1.9 (-31.4−50.9) | -4.0 (-37.2−47.9) | | | 0.306 |
| **CD19^+^CD20^+^CD27^+^** | 129.2 (60.0−242.6) | 120.0 (56.0−237.1) | | | 0.366 |
| **CD19^+^CD20^+^CD69^+^** | -14.4 (-68.6−109.1) | -16.5 (-61.2−100.4) | | | 0.548 |
| **CD19^+^CD20^+^CD27^-^** | -28.6 (-50.9−12.8) | -28.6 (-52.7−10.5) | | | 0.546 |
| **CD19^+^CD20^+^CD138^+^** | -9.5 (-68.5−114.9) | -24.4 (-68.1−78.2) | | | 0.279 |
| **CD19^+^CD20^-^CD138^+^** | -29.1 (-70.8−51.0) | -27.9 (-69.0−57.6) | | | 0.789 |
| **CD19^+^CD20^-^CD27^brt^** | -37.7 (-71.4−0.0) | -42.9 (-74.9−8.0) | | | 0.572 |
| **CD19^+^CD27^brt^CD38^brt^** | -32.7 (-64.0−35.7) | -37.5 (-67.6−32.5) | | | 0.465 |
| **Serological markers** |  |  | | |  |
| **C3** | 4.9 (-5.9−17.2) | 2.1 (-8.7−14.7) | | | **0.014** |
| **C4** | 11.5 (0.0−31.8) | 8.3 (-6.3−27.4) | | | **0.017** |
| **anti-dsDNA** (all patients) | -14.8 (-37.7−0.0) | -8.7 (-32.2−0.0) | | | **0.043** |
| **anti-dsDNA** (patients positive at baseline) | -24.7 (-43.8−-2.7) | -21.1 (-39.6−0.2) | | | 0.083 |
| **Placebo** | | | | | |
| **B cell subsets** |  |  | | |  |
| **CD19^+^CD20^+^** | 0.0 (-19.7−32.8) | -7.8 (-27.2−23.2) | | | 0.065 |
| **CD19^+^CD20^+^CD27^+^** | 0.0 (-27.2−35.1) | -7.1 (-35.0−37.5) | | | 0.098 |
| **CD19^+^CD20^+^CD69^+^** | -5.8 (-68.4−108.4) | -22.8 (-66.9−117.6) | | | 0.484 |
| **CD19^+^CD20^+^CD27^-^** | 0.0 (-22.3−35.6) | -6.7 (-26.6−24.1) | | | 0.082 |
| **CD19^+^CD20^+^CD138^+^** | -19.0 (-64.5−94.5) | -17.4 (-72.5−86.7) | | | 0.581 |
| **CD19^+^CD20^-^CD138^+^** | -12.6 (-61.9−67.2) | -16.0 (-60.0−78.2) | | | 0.824 |
| **CD19^+^CD20^-^CD27^brt^** | -18.2 (-50.0−48.0) | -2.7 (-55.8−65.0) | | | 0.317 |
| **CD19^+^CD27^brt^CD38^brt^** | -1.6 (-50.7−82.0) | -2.3 (-53.0−88.1) | | | 0.969 |
| **Serological markers** |  |  | | |  |
| **C3** | 0.0 (-9.5−11.5) | 0.0 (-10.7−11.9) | | | 0.821 |
| **C4** | 0.0 (-11.1−17.8) | 0.0 (-13.1−16.7) | | | 0.425 |
| **anti-dsDNA** (all patients) | 0.0 (-23.9−8.6) | 0.0 (-21.5−11.8) | | | 0.395 |
| **anti-dsDNA** (patients positive at baseline) | -9.2 (-33.1−13.1) | -3.4 (-28−19.5) | | | 0.223 |
| **Belimumab vs placebo** (P value) | | | | | |
| **B cell subsets** |  |  | | |  |
| **CD19^+^CD20^+^** | 0.676 | 0.766 | | | N/A |
| **CD19^+^CD20^+^CD27^+^** | **<0.001** | **<0.001** | | | N/A |
| **CD19^+^CD20^+^CD69^+^** | 0.529 | 0.455 | | | N/A |
| **CD19^+^CD20^+^CD27^-^** | **<0.001** | **<0.001** | | | N/A |
| **CD19^+^CD20^+^CD138^+^** | 0.785 | 0.905 | | | N/A |
| **CD19^+^CD20^-^CD138^+^** | 0.077 | **0.038** | | | N/A |
| **CD19^+^CD20^-^CD27^brt^** | **0.001** | **<0.001** | | | N/A |
| **CD19^+^CD27^brt^CD38^brt^** | **<0.001** | **<0.001** | | | N/A |
| **Serological markers** |  |  | | |  |
| **C3** | **0.001** | 0.072 | | | N/A |
| **C4** | **<0.001** | **<0.001** | | | N/A |
| **anti-dsDNA** (all patients) | **<0.001** | **<0.001** | | | N/A |
| **anti-dsDNA** (patients positive at baseline) | **<0.001** | **<0.001** | | | N/A |

Data are presented as medians (interquartile range) of the relative to baseline percentage changes. P values are derived from non-parametrical Mann-Whitney *U* tests. Statistically significant P values are in bold.

C3: complement component 3; C4: complement component 4; N/A: not applicable; SRI-4: Systemic Lupus Erythematosus Responder Index 4.

**Supplementary Table S2.** Relative to baseline percentage changes through week 24 in B cell subset counts and serum levels of serological markers in SRI-4 responders versus non-responders at week 52 in the pooled BLISS study population.

|  | **SRI-4** | | **No SRI-4** | **P value** | |
| --- | --- | --- | --- | --- | --- |
| **Entire patient cohort** (all treatment arms) | | | | | |
| **B cell subsets** |  | |  |  | |
| **CD19^+^CD20^+^** | -21.2 (-51.2−23.5) | -18.8 (-48.4−19.5) | | | 0.836 |
| **CD19^+^CD20^+^CD27^+^** | 60.0 (-5.4−166.1) | 49.5 (-11.1−126.9) | | | **0.033** |
| **CD19^+^CD20^+^CD69^+^** | -31.0 (-73.6−75.0) | -32.1 (-72.5−83.1) | | | 0.770 |
| **CD19^+^CD20^+^CD27^-^** | -42.5 (-66.3−0.0) | -33.5 (-64.5−1.2) | | | 0.163 |
| **CD19^+^CD20^+^CD138^+^** | -37.5 (-77.5−66.9) | -41.9 (-76.3−56.3) | | | 0.858 |
| **CD19^+^CD20^-^CD138^+^** | -40.0 (-77.9−46.7) | -33.9 (-73.3−38.6) | | | 0.174 |
| **CD19^+^CD20^-^CD27^brt^** | -38.9 (-75.0−5.9) | -34.3 (-71.4−17.5) | | | 0.259 |
| **CD19^+^CD27^brt^CD38^brt^** | -29.2 (-66.2−35.3) | -24.8 (-65.7−59.9) | | | 0.205 |
| **Serological markers** |  |  | | |  |
| **C3** | 4.0 (-7.4−16.8) | 1.3 (-9.3−16.2) | | | 0.103 |
| **C4** | 12.5 (-2.9−36.4) | 10.0 (-7.8−33.3) | | | **0.017** |
| **anti-dsDNA** (all patients) | -21.8 (-48.5−0.0) | 0.0 (-34.5−2.7) | | | **<0.001** |
| **anti-dsDNA** (patients positive at baseline) | -34.8 (-55.5−-6.5) | -20.3 (-44.3−13.2) | | | **<0.001** |
| **Belimumab** | | | | | |
| **B cell subsets** |  |  | | |  |
| **CD19^+^CD20^+^** | -29.2 (-57.2−16.9) | -32.1 (-60.0−11.1) | | | 0.393 |
| **CD19^+^CD20^+^CD27^+^** | 95.1 (18.1−200.0) | 83.6 (21.2−171.2) | | | 0.162 |
| **CD19^+^CD20^+^CD69^+^** | -31.4 (-73.8−72.6) | -37.0 (-73.0−87.2) | | | 0.968 |
| **CD19^+^CD20^+^CD27^-^** | -53.6 (-70.9−-22.3) | -55.1 (-73.5−-25.8) | | | 0.604 |
| **CD19^+^CD20^+^CD138^+^** | -41.9 (-80.7−45.1) | -51.6 (-78.8−42.3) | | | 0.621 |
| **CD19^+^CD20^-^CD138^+^** | -43.1 (-80.0−41.0) | -44.9 (-77.1−19.5) | | | 0.894 |
| **CD19^+^CD20^-^CD27^brt^** | -50.0 (-77.1−0.0) | -47.9 (-77.1−1.2) | | | 0.634 |
| **CD19^+^CD27^brt^CD38^brt^** | -40.1 (-69.1−17.0) | -36.4 (-71.1−35.0) | | | 0.796 |
| **Serological markers** |  |  | | |  |
| **C3** | 5.5 (-4.9−18.7) | 3.0 (-8.1−16.7) | | | 0.101 |
| **C4** | 15.9 (0.0−42.9) | 13.3 (0.0−37.5) | | | **0.049** |
| **anti-dsDNA** (all patients) | -26.8 (-52.2−0.0) | -9.0 (-41.7−0.0) | | | **<0.001** |
| **anti-dsDNA** (patients positive at baseline) | -40.9 (-58.3−-13.5) | -26.9 (-48.5−0.0) | | | **<0.001** |
| **Placebo** | | | | | |
| **B cell subsets** |  |  | | |  |
| **CD19^+^CD20^+^** | -1.1 (-33.2−33.3) | -5.6 (-28.1−30.2) | | | 0.912 |
| **CD19^+^CD20^+^CD27^+^** | -9.1 (-38.9−50.0) | 0.0 (-33.3−51.0) | | | 0.421 |
| **CD19^+^CD20^+^CD69^+^** | -30.3 (-70.0−81.2) | -25.0 (-70.0−70.0) | | | 0.766 |
| **CD19^+^CD20^+^CD27^-^** | 0.0 (-35.7−37.5) | -5.5 (-29.3−32.0) | | | 0.801 |
| **CD19^+^CD20^+^CD138^+^** | -21.9 (-69.2−86.6) | -18.9 (-71.6−84.6) | | | 0.872 |
| **CD19^+^CD20^-^CD138^+^** | -32.8 (-71.1−62.2) | -14.8 (-63.6−57.6) | | | 0.108 |
| **CD19^+^CD20^-^CD27^brt^** | -12.0 (-55.1−50.0) | -14.2 (-54.8−50.0) | | | 0.696 |
| **CD19^+^CD27^brt^CD38^brt^** | -4.2 (-48.7−83.8) | -2.2 (-52.5−102.8) | | | 0.471 |
| **Serological markers** |  |  | | |  |
| **C3** | -0.9 (-10.7−9.4) | -1.3 (-11.8−14.5) | | | 0.852 |
| **C4** | 0.0 (-12.9−20.0) | 1.7 (-12.5−25.0) | | | 0.925 |
| **anti-dsDNA** (all patients) | -4.3 (-34.9−0.0) | 0.0 (-24.6−23.2) | | | **<0.001** |
| **anti-dsDNA** (patients positive at baseline) | -25.1 (-46.8−1.2) | -5.3 (-31.6−28.6) | | | **<0.001** |
| **Belimumab vs placebo** (P value) | | | | | |
| **B cell subsets** |  |  | | |  |
| **CD19^+^CD20^+^** | **<0.001** | **<0.001** | | | N/A |
| **CD19^+^CD20^+^CD27^+^** | **<0.001** | **<0.001** | | | N/A |
| **CD19^+^CD20^+^CD69^+^** | 0.608 | 0.403 | | | N/A |
| **CD19^+^CD20^+^CD27^-^** | **<0.001** | **<0.001** | | | N/A |
| **CD19^+^CD20^+^CD138^+^** | **0.014** | **0.004** | | | N/A |
| **CD19^+^CD20^-^CD138^+^** | 0.167 | **<0.001** | | | N/A |
| **CD19^+^CD20^-^CD27^brt^** | **<0.001** | **<0.001** | | | N/A |
| **CD19^+^CD27^brt^CD38^brt^** | **<0.001** | **<0.001** | | | N/A |
| **Serological markers** |  |  | | |  |
| **C3** | **<0.001** | **0.020** | | | N/A |
| **C4** | **<0.001** | **<0.001** | | | N/A |
| **anti-dsDNA** (all patients) | **<0.001** | **<0.001** | | | N/A |
| **anti-dsDNA** (patients positive at baseline) | **<0.001** | **<0.001** | | | N/A |

Data are presented as medians (interquartile range) of the relative to baseline percentage changes. P values are derived from non-parametrical Mann-Whitney *U* tests. Statistically significant P values are in bold.

C3: complement component 3; C4: complement component 4; N/A: not applicable; SRI-4: Systemic Lupus Erythematosus Responder Index 4.

**Supplementary Table S3.** Relative to baseline percentage changes through week 52 in B cell subset counts and serum levels of serological markers in SRI-4 responders versus non-responders at week 52 in the pooled BLISS study population.

|  | **SRI-4** | **No SRI-4** | | | **P value** |
| --- | --- | --- | --- | --- | --- |
| **Entire patient cohort** (all treatment arms) | | | | | |
| **B cell subsets** |  | |  |  | |
| **CD19^+^CD20^+^** | -43.8 (-66.8−-3.6) | -34.7 (-63.1−6.2) | | | **0.023** |
| **CD19^+^CD20^+^CD27^+^** | 14.3 (-28.6−97.7) | 16.7 (-26.9−90.9) | | | 0.988 |
| **CD19^+^CD20^+^CD69^+^** | -43.0 (-80.8−44.0) | -34.4 (-76.8−41.6) | | | 0.300 |
| **CD19^+^CD20^+^CD27^-^** | -61.2 (-78.8−-18.5) | -50.0 (-74.8−-8.3) | | | **0.004** |
| **CD19^+^CD20^+^CD138^+^** | -45.6 (-81.1−41.1) | -54.3 (-82.3−23.6) | | | 0.353 |
| **CD19^+^CD20^-^CD138^+^** | -48.2 (-80.3−29.8) | -37.1 (-74.4−49.5) | | | **0.024** |
| **CD19^+^CD20^-^CD27^brt^** | -44.9 (-78.3−0.0) | -33.3 (-75.0−43.0) | | | **0.011** |
| **CD19^+^CD27^brt^CD38^brt^** | -38.9 (-71.4−30.8) | -28.9 (-72.3−46.5) | | | 0.148 |
| **Serological markers** |  |  | | |  |
| **C3** | 6.3 (-5.2−21.8) | 0.0 (-10.2−13.3) | | | **<0.001** |
| **C4** | 18.2 (0.0−44.4) | 10.0 (-11.1−33.3) | | | **<0.001** |
| **anti-dsDNA** (all patients) | -34.8 (-64.0−0.0) | -2.0 (-42.6−0.0) | | | **<0.001** |
| **anti-dsDNA** (patients positive at baseline) | -48.7 (-69.5−-21.2) | -28.3 (-51.8−7.5) | | | **<0.001** |
| **Belimumab** | | | | | |
| **B cell subsets** |  |  | | |  |
| **CD19^+^CD20^+^** | -54.5 (-72.2—20.0) | -50.0 (-71.2−-9.8) | | | 0.194 |
| **CD19^+^CD20^+^CD27^+^** | 27.8 (-17.5−115.0) | 45.5 (-8.9−121.8) | | | 0.235 |
| **CD19^+^CD20^+^CD69^+^** | -44.8 (-81.4−41.3) | -41.7 (-80.8−37.1) | | | 0.673 |
| **CD19^+^CD20^+^CD27^-^** | -70.3 (-82.3—50.0) | -67.7 (-82.7−-45.1) | | | 0.168 |
| **CD19^+^CD20^+^CD138^+^** | -52.8 (-82.1−20.3) | -60.4 (-84.4−-0.8) | | | 0.138 |
| **CD19^+^CD20^-^CD138^+^** | -51.3 (-82.5−19.1) | -44.4 (-77.9−40.1) | | | 0.104 |
| **CD19^+^CD20^-^CD27^brt^** | -50.0 (-84.3−0.0) | -50.0 (-83.0−17.7) | | | 0.251 |
| **CD19^+^CD27^brt^CD38^brt^** | -47.1 (-73.7−19.1) | -44.5 (-77.6−36.9) | | | 0.967 |
| **Serological markers** |  |  | | |  |
| **C3** | 7.9 (-2.8−26.3) | 3.0 (-8.9−17.9) | | | **<0.001** |
| **C4** | 23.5 (5.0−50.7) | 15.6 (0.0−38.8) | | | **<0.001** |
| **anti-dsDNA** (all patients) | -39.2 (-65.6−0.0) | -16.7 (-50.5−0.0) | | | **<0.001** |
| **anti-dsDNA** (patients positive at baseline) | -51.5 (-71.1−-26.7) | -38.3 (-61.2−-12.6) | | | **<0.001** |
| **Placebo** | | | | | |
| **B cell subsets** |  |  | | |  |
| **CD19^+^CD20^+^** | -8.4 (-42.2−31.1) | -12.5 (-39.6−32.5) | | | 0.883 |
| **CD19^+^CD20^+^CD27^+^** | -10.9 (-46.3−51.4) | -14.3 (-43.1−50.0) | | | 0.866 |
| **CD19^+^CD20^+^CD69^+^** | -40.3 (-75.0−58.0) | -16.5 (-71.2−59.8) | | | 0.391 |
| **CD19^+^CD20^+^CD27^-^** | -10.5 (-40.8−31.5) | -10.3 (-40.0−32.4) | | | 0.841 |
| **CD19^+^CD20^+^CD138^+^** | -23.8 (-78.0−78.0) | -32.7 (-78.2−72.3) | | | 0.880 |
| **CD19^+^CD20^-^CD138^+^** | -38.1 (-72.0−76.6) | -21.3 (-69.1−80.2) | | | 0.348 |
| **CD19^+^CD20^-^CD27^brt^** | -29.3 (-59.6−39.1) | 0.0 (-50.0−83.3) | | | **0.032** |
| **CD19^+^CD27^brt^CD38^brt^** | -20.1 (-59.9−79.5) | -0.7 (-49.3−101.6) | | | 0.137 |
| **Serological markers** |  |  | | |  |
| **C3** | 1.3 (-11.1−14.2) | -3.2 (-12.1−9.3) | | | **0.043** |
| **C4** | 5.0 (-8.6−25.0) | 0.0 (-20.0−16.7) | | | **<0.001** |
| **anti-dsDNA** (all patients) | -16.2 (-51.8−0.0) | 0.0 (-21.6−22.5) | | | **<0.001** |
| **anti-dsDNA** (patients positive at baseline) | -39.3 (-63.8−-1.9) | -7.1 (-34.1−28.6) | | | **<0.001** |
| **Belimumab vs placebo** (P value) | | | | | |
| **B cell subsets** |  |  | | |  |
| **CD19^+^CD20^+^** | **<0.001** | **<0.001** | | | N/A |
| **CD19^+^CD20^+^CD27^+^** | **<0.001** | **<0.001** | | | N/A |
| **CD19^+^CD20^+^CD69^+^** | 0.135 | **0.050** | | | N/A |
| **CD19^+^CD20^+^CD27^-^** | **<0.001** | **<0.001** | | | N/A |
| **CD19^+^CD20^+^CD138^+^** | **0.004** | **0.001** | | | N/A |
| **CD19^+^CD20^-^CD138^+^** | **0.004** | **0.011** | | | N/A |
| **CD19^+^CD20^-^CD27^brt^** | **<0.001** | **<0.001** | | | N/A |
| **CD19^+^CD27^brt^CD38^brt^** | **<0.001** | **<0.001** | | | N/A |
| **Serological markers** |  |  | | |  |
| **C3** | **<0.001** | **<0.001** | | | N/A |
| **C4** | **<0.001** | **<0.001** | | | N/A |
| **anti-dsDNA** (all patients) | **<0.001** | **<0.001** | | | N/A |
| **anti-dsDNA** (patients positive at baseline) | **<0.001** | **<0.001** | | | N/A |

Data are presented as medians (interquartile range) of the relative to baseline percentage changes. P values are derived from non-parametrical Mann-Whitney *U* tests. Statistically significant P values are in bold.

C3: complement component 3; C4: complement component 4; N/A: not applicable; SRI-4: Systemic Lupus Erythematosus Responder Index 4.
